# Verhalten verstehen, um Gesundheit für alle zu ermöglichen: Der Ansatz „Behavioural and Cultural Insights“

**DOI:** 10.1007/s00103-025-04112-7

**Published:** 2025-08-27

**Authors:** Julika Loss, Anke-Christine Saß, Cornelia Betsch

**Affiliations:** 1 Abteilung für Epidemiologie und Gesundheitsmonitoring, Robert Koch-Institut, Gerichtstr. 27, 13347 Berlin, Deutschland; 2 Institute for Planetary Health Behaviour (IPB), Universität Erfurt, Max-Weber-Allee 3, 99089 Erfurt, Deutschland; 3 Health Communication Working Group, Implementation Science, Berhnard-Nocht-Institut für Tropenmedizin, Bernhard-Nocht-Str. 74, 20359 Hamburg, Deutschland

Prävention stellt einen entscheidenden Handlungsansatz für Public Health dar, da viele Volkskrankheiten vermeidbar sind. In den letzten Jahrzehnten haben sich grundlegende Prinzipien der Prävention etabliert. Dazu gehört insbesondere, nicht allein das individuelle gesundheitsbezogene Verhalten in den Blick zu nehmen, sondern auch die sozialen und kulturellen Rahmenbedingungen, die Verhalten ermöglichen oder erschweren. Studien zu sozial bedingter Ungleichheit zeigen, dass gesundheitsbezogenes Verhalten in der Bevölkerung je nach Geschlecht, Alter, Einkommen, Bildung und Kultur variiert. Eine universelle Lösung im Sinne eines „One-size-fits-all“-Ansatzes für Prävention kann es also nicht geben, hingegen kann das Betrachten dieser Aspekte für mehr Gesundheitsgerechtigkeit sorgen.

Präventive Ansätze müssen demnach an die Bedürfnisse, Bedarfe und Lebensbedingungen von denjenigen Bevölkerungsgruppen angepasst sein, die man erreichen möchte. „Tailoring health programmes“ nennt die Weltgesundheitsorganisation (WHO) das, also das „Maßschneidern“ von gesundheitsbezogenen Interventionen. Was es dazu braucht? Zum einen ein Verständnis dessen, welche Faktoren es bestimmten Bevölkerungsgruppen erschweren, sich im Alltag gesundheitsförderlich zu verhalten – oder was es ihnen erleichtert. Warum bewegen sich Studierende auf dem Campus so wenig? Was beeinflusst die Impfentscheidung von Eltern? Wie kann es gelingen, dass sich Berufstätige gesünder ernähren? Zum anderen muss man Maßnahmen, die aufbauend auf diesen Erkenntnissen entwickelt werden, hinsichtlich ihrer Wirksamkeit untersuchen – um weitere Hinweise zu gewinnen, welcher präventive Ansatz in welcher Gruppe effektiv ist.

Das Konzept der „Behavioural and Cultural Insights“, abgekürzt BCI, versucht genau das: empirische Erkenntnisse (Insights) zu gewinnen, die helfen zu verstehen, warum sich Menschen in bestimmten Situationen gesundheitsförderlich verhalten oder eben auch nicht. Umgebungsfaktoren und kulturelle Kontexte sollen dabei explizit in den Blick genommen werden. Die gewonnenen Erkenntnisse sollen gezielt für die Gestaltung gesundheitspolitischer Maßnahmen und begleitender Kommunikation genutzt werden. Wie wirksam diese Maßnahmen sind, soll durch Evaluationen untersucht werden. Damit orientiert sich das BCI-Konzept am „Public Health Action Cycle“, der dem Kreislauf „Plan-Do-Check-Act“ folgt (siehe auch den Beitrag von *Ewert* in diesem Heft).

„Behavioural and cultural insights“ beziehen sich jeweils auf ein bestimmtes Verhalten – zum Beispiel Alkoholkonsum bei Heranwachsenden oder Inanspruchnahme von Impfungen durch eine besondere ethnische Community. Auch wenn das Verhalten im Fokus steht – die Perspektive ist nicht auf die individuelle Verhaltensänderung bzw. die Verhaltensprävention beschränkt. Im Gegenteil: BCI tragen dazu bei, das Bewusstsein für den Zusammenhang zwischen Verhalten und Umwelt zu schärfen, und bieten die Möglichkeit, Verhältnis- und Verhaltensprävention besser zu verbinden. Das zeigt auch das von *Loss et al.* vorgestellte Projekt: Das Forschungsteam hat Barrieren für Bewegung von Studierenden identifiziert, die mehrheitlich in den Eigenschaften des Uni-Geländes und der Struktur des Hochschulalltags begründet sind. Entsprechend wurden Interventionen entwickelt, die die Rahmenbedingungen für körperliche Aktivität an den beteiligten Universitäten verbessern. Auch *Pedrós-Barnils et al.*, die sich in ihrem Beitrag mit BCI und Ernährungsverhalten befassen, heben hervor: Um soziale Unterschiede in der Ernährungsqualität zu verringern, sind Strategien sowohl auf struktureller als auch individueller Ebene notwendig, zum Beispiel Werbebeschränkungen für ungesunde Lebensmittel und frühzeitige Bildungsangebote zur Ernährung.

BCI, wie wir sie heute verstehen und anwenden, betrachten strukturelle, individuelle und motivationale Einflussfaktoren gleichwertig. Das Konzept hat allerdings auch Wurzeln in der Verhaltensökonomie. Diese geht davon aus, dass Impulsivität, soziale Normen und Reaktionen auf Schlüsselreize unser Verhalten steuern. Der Ansatz des „Nudging“ soll diese impulsive, nicht unbedingt rational gesteuerte Entscheidungsfindung beeinflussen. Obgleich Nudging-Ansätze auch für Public Health sinnvoll sein können, repräsentieren sie nur einen kleinen Teil von möglichen Interventionsansätzen, die auf BCI beruhen. Zwei Beiträge in diesem Heft untersuchen die Wirksamkeit von Nudging für Public-Health-Belange: *Rebitschek et al.* vergleichen für das Thema Impfentscheidung die Wirkung von Nudging unter anderem mit der von evidenzbasierten Faktenboxen; *Meis-Harris et al*. analysieren die Wirksamkeit von Nudging-Interventionen, die nachhaltiges Ernährungsverhalten in der Gemeinschaftsverpflegung verbessern sollen.

Das WHO-Regionalbüro für Europa hat das Thema BCI als ein „Flaggschiff“ ihres Arbeitsprogrammes 2020–2025 bezeichnet und 2022 die Resolution „European regional action framework for behavioural and cultural insights for equitable health“ verabschiedet. Dabei wurden gesundheitliche Chancengleichheit als Ziel von BCI sowie die kulturelle Dimension von Gesundheit in den Mittelpunkt gerückt. Hierum geht es im Artikel von *Snijders et al*. Laut den Autorinnen und Autoren ist es entscheidend zu verstehen, wie kulturelle Kontexte unser Gesundheitsverhalten beeinflussen – z. B. die Zugehörigkeit zu einer Religionsgemeinschaft, einer Nationalität oder einer ländlichen versus urbanen Nachbarschaft. Präventive Maßnahmen sind entsprechend kulturell zu verankern, da Gesundheitsgerechtigkeit eng mit kulturellen Rahmenbedingungen verflochten ist.

Der BCI-Ansatz basiert auf einem empirischen Vorgehen. Die Sammlung von Daten, die helfen, menschliches Verhalten zu verstehen, ist ein entscheidender Bestandteil von BCI. Wie eine geeignete Datenerhebung aussehen kann, legen *Daube et al*. in ihrem Beitrag dar. Online-Umfragen und Experience Sampling in großen Stichproben, aber auch qualitative Methoden (z. B. Interviews, Tagebuchstudien) können Verhaltensmuster aufdecken, Entscheidungen nachzeichnen und soziale sowie psychologische Einflüsse identifizieren. Eine innovative Methode der Datengewinnung für BCI berichten *Zintel et al.; *sie stellen den Aufbau einer agilen Kohorte vor, die Echtzeitinformationen zu Gesundheitsverhalten und kontextuellen Bedingungen hochauflösend erfassen soll. Vielschichtige Verhaltensdaten im Sinne von Behavioural and Cultural Insights liefern ein klareres Bild darüber, wem es wann, wo und aus welchen Gründen gelingt, sich gesundheitsfördernd zu verhalten. Sie schaffen die Grundlage für Interventionen, die Verhaltens- und Verhältnisprävention gemeinsam adressieren – über verhaltensökonomische Nudging-Ansätze hinaus, durch Kompetenzförderung, kulturelle Sensibilität und Schaffung unterstützender Lebensverhältnisse.

Die Artikel zeigen, dass BCI für viele Public-Health-Themen relevante Impulse geben und Aktivitäten anstoßen kann – zu Ernährung, Bewegung, Klimaschutz oder Impfverhalten. BCI ist dabei kein komplett neuer Ansatz, sondern weist viele Schnittstellen und Übereinstimmungen mit Prinzipien für die Gestaltung von evidenzbasierten und bedarfsorientierten gesundheitsbezogenen Interventionen und Kampagnen auf. Kernmerkmale von BCI sind die systematische Erhebung und Anwendung von Daten, die das menschliche Verhalten in Gesundheitsbelangen erhellen können.

Trotz der im Heft versammelten ermutigenden Beiträge: BCI-Aktivitäten werden in Deutschland bislang nicht systematisch gefördert, angewandt oder koordiniert. Dadurch bleibt das Potenzial von BCI, zur Verbesserung der Bevölkerungsgesundheit und Gesundheitsgerechtigkeit beizutragen, politisch bislang weitgehend ungenutzt. Wie es gelingen kann, zeigt ein Blick nach Wales: Dort arbeitet eine eigene Fachabteilung des Public Health Instituts – die Behavioural Science Unit of Public Health Wales – daran, BCI routinemäßig und systematisch zur Förderung der Gesundheit sowie zum Abbau gesundheitlicher Ungleichheiten zu nutzen, wie *Gould* beschreibt.

Das vorliegende Themenheft soll Mut machen, BCI weiter auszubauen und als wichtige Säule für Public-Health- und Präventionsmaßnahmen zu nutzen.

Wir wünschen allen Leserinnen und Lesern eine anregende Lektüre und Impulse für gelingende Prävention.

